# An Active Cooperation-Aware Spectrum Allocation Mechanism for Body Sensor Networks

**DOI:** 10.3390/s150202812

**Published:** 2015-01-28

**Authors:** Fu Jiang, Ying Guo, Jun Peng, Jiankun Hu

**Affiliations:** 1 School of Information Science and Engineering, Central South University, Changsha 410075, China; E-Mails: jiangfu0912@csu.edu.cn (F.J.); peng.pengj@gmail.com (J.P.); 2 School of Engineering and Information Technology, The University of New South Wales at the Australian Defense Force Academy, Canberra ACT 2600, Australia; E-Mail: J.Hu@adfa.edu.au

**Keywords:** body sensor networks, cognitive radio networks, cooperative communication, active cooperation, Stackelberg game, resource allocation

## Abstract

A cognitive radio-based spectrum allocation scheme using an active cooperative-aware mechanism is proposed in this paper. The scheme ensures that the primary user and secondary users cooperate actively for their own benefits. The primary user releases some spectrum resources to secondary users to actively stimulate them to actively join the cooperative transmission of the primary user, and secondary users help the primary user to relay data in return, as well as its self-data transmission at the same time. The Stackelberg game is used to evenly and jointly optimize the utilities of both the primary and secondary users. Simulation results show that the proposed active cooperation-aware mechanism could improve the body sensor network performance.

## 1. Introduction

Recently, the applications of wireless body sensor networks have grown considerably. In body sensor networks, tiny sensor nodes are worn on or implanted into human body to detect physical signals such as temperature, blood pressure, heart-rate, motion, *etc.* The body sensors are typically deployed with higher density and more limited resources than in general wireless sensor networks. Its concurrent data transmission can be hindered if there is not efficient spectrum management and power control [1]. Subsequently, body sensor networks have emerged as a powerful tool in medical care due to their capability of collecting health data in real-time [2]. A body sensor network consists of many tiny sensors which are used to monitor health data from the body. One typical characteristic of a body sensor network is its very limited transmission power, as these tiny sensors are running continuously in a 24/7 mode [3], therefore energy-efficient relay transmission is very important for the body sensor networks. One unique characteristic of the body sensor networks is that these sensors will transmit multiple different body signals, e.g., heart beat rate and blood pressure, *etc.*, concurrently, which will make spectrum management very challenging.

Cognitive radio is a powerful tool for dynamic spectrum management [4] that can greatly improve the spectrum efficiency [5,6]. Its basic idea is allowing secondary users (SUs) to coexist with primary users (PUs) in the same spectrum by using spectrum access technology [7]. This coexistence requires an agreement between primary and secondary users on a spectrum access strategy. The traditional agreement in [8] assumes that primary users have no idea about the existence of secondary users, and secondary users use opportunities to access the spectrum only when it is not used by primary users.

Unfortunately in a body sensor network, the primary user/sensor needs these secondary users/sensors as relay nodes and these relay nodes also need to transmit their data at the same time. Therefore cooperative communication is needed in such environment. Several schemes have been proposed to study cooperative communications among a primary user and secondary users in order to improve secondary users' transmission rate. In reference [9], under the assumption that the SUs know perfectly the information of the primary user, the SUs transmit the data of both the SUs and the primary user over the spectrum of the primary user simultaneously by jointly encoding their data, thus improving the overall transmission rate. In reference [10], the author proposed a more realistic scheme where secondary users only forward the primary user's unsuccessful data packets in the spectrum holes at the same time by using dirty-paper coding [11]. However, secondary users also need a more continuous quality of their communication, as they will be interrupted frequently when the primary user is busy, and the continuity and quality of secondary users' communication cannot be ensured, besides the selfless secondary users mentioned above must know everything about the primary user which is unrealistic in a body sensor network environment.

The active cooperation mechanism [12] is a new cooperative communication approach which considers the cooperation between primary user and secondary users. It takes secondary users as relay nodes that help forward the primary user's signals in exchange for unused spectrum and the spectrum released by the primary user will be used by secondary users for their own data transmission. It is backed by comprehensive research and could solve many practical problems. There are several existing research achievements regarding the active cooperation mechanism. In [12,13], the active cooperative mechanism is used in a cognitive *ad-hoc* environment scenario to allow secondary users to obtain a certain opportunity to access the channel. Thus secondary users could maintain a continuous reliable communication. The authors in reference [14] propose a pricing-based active cooperation framework, where the primary user maximizes its utility by setting the spectrum price and the selected secondary users decide their power levels to help the primary user's transmission, aiming at obtaining a corresponding spectrum access time.

Based on these works, the different priorities and selfishness of both the primary user and secondary users have been taken into consideration in this paper, and an improved active cooperation mechanism which consists of one primary user and multiple secondary users is proposed for cognitive radio networks. A primary user actively chooses some suitable secondary users and stimulates them as cooperative relays by giving secondary users a portion of the available spectrum. Secondary users could in turn transmit their data over the spectrum released by the primary user. Thus, secondary users should help primary user's cooperative transmission and meanwhile pay charges to the primary user in order to access the released spectrum for their own transmissions.

It is assumed that both the primary user and secondary users are selfish and rational network users, which means all of them are only interested in optimizing each one's own profits:

1. For the primary user, the main objective is to maximize its utility, including the primary user's transmission rate and the extra revenue achieved from secondary users.
2. For secondary users, the target is to maximize their transmission rate by paying the primary user as little as possible.

In order to achieve these goals, a satisfactory function for the primary user to differ the relay capability of secondary users is derived. Then game theory is employed used to describe and analyze this framework. Game theory is a mature mathematical tool that could be used to study the complex interactions among interdependent rational players [15]. It plays an important role in many fields [16,17]. In the past decade, game theory has been used to describe and analyze the competition and cooperation among users in wireless cognitive radio networks [15,18].

In addition, the proposed active cooperation scenario is characterized by a hierarchical architecture, where the primary user has the priority to decide the game's strategy. Then, secondary users react to the primary user's strategy, which means secondary users optimize their strategies based on their knowledge of the effects of their decisions on the behavior of the primary user. Therefore, in this paper, the active cooperation framework is modeled as a classical two level leader-follower Stackelberg game [19,20]. This approach could distinguish the priorities of the primary user and the secondary users by modeling them as leader and followers, respectively. To solve the game, the backward induction method is used to prove the existence and uniqueness of the Nash equilibrium. Thus, the primary user could improve the communication performance and achieve extra revenue as much as possible, while the secondary users obtain the sustaining opportunity to access the authorized spectrum by jointly cooperating and paying some reasonable charge, so that a win-win situation for leader and followers in the game can be achieved.

The rest of this paper is organized as follows: the system model and the problem formulation are discussed in Section 2. Game-theory analysis and the optimal solution of the proposed model are presented in Section 3. Numerical results are presented in Section 4. Finally the work is summarized and concluding remarks are offered in Section 5.

## 2. Active Cooperation-Aware System Model and Problem Formulation

### 2.1. System Model

The proposed body sensor network is composed by two types of node. One is implant nodes, which use the licensed 402–405 MHz band spectrum to transmit data. The spectrum has been licensed for the medical implant communications service (MICS). Another is emerging wearable nodes, which use the ISM spectrum, such as Wi-Fi or Zigbee, to transmit data. The ISM spectrum could be jammed by other electronic devices due to its free property. Thus, in the paper, to ensure the transmission reliability for wearable nodes, they can be taken as cognitive users that can utilize the licensed MICS spectrum if they do not affect the primary users' transmission.

**Figure 1.**
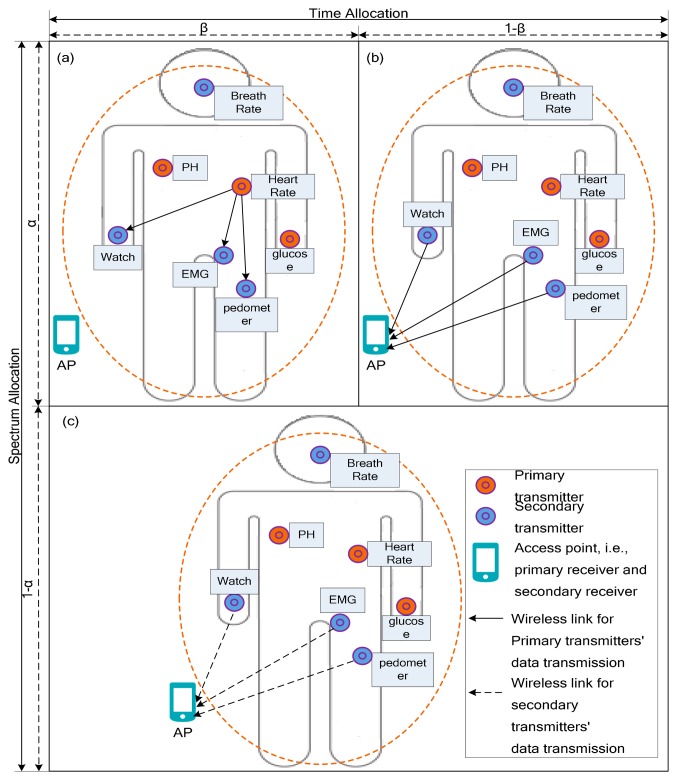
The time-spectrum allocation of primary user and secondary users in active cooperation mechanism: (**a**) in fraction *αβ* of the time-spectrum slot, PT broadcast data to ST*_i_* in selected secondary subset *S*; (**b**) in fraction *α*(1 − *β*) of time-spectrum slot, all ST*_i_* cooperatively transmit primary data to PR; (**c**) in fraction *α* of spectrum slot, ST*_i_* transmit their own data to SR.

The system is presented in Figure 1, where there are several primary transmitters (PT), the implant sensor nodes, *i.e.*, PH sensor, heart rate sensor and glucose sensor. The PT communicates with the primary receiver (PR) using the licensed spectrum. The system also has K secondary transmitters (ST) and a secondary receiver (SR), which are wearable nodes using the ISM spectrum, but they can sense the licensed spectrum for implant nodes and use it when the licensed spectrum is idle.

On the other hand, due to the resource limitations of the implant nodes, they have more restricted power requirements. When there is long distance between the primary transmitter and the primary receiver, the directed transmission will lead to the implant node increasing its sending power and consequently, shorting its lifetime. If the primary user can actively lease some its licensed spectrum to stimulate the secondary users to help with its transmission, its power efficiency will be improved.

It is assumed that the secondary users can be denoted by ${\left\{ST_{i},\;SR_{i}\right\}}_{i=1}^{K}$ . They are seeking to exploit possible transmission resources. The primary transmitters actively choose the sensor relays set S which is composed of *k* secondary users, where |*S*| = *k* ≤ |*S_total_*| = *K*. The PT grants the use of the spectrum to the secondary node subset *S* in exchange for cooperation so as to improve the communication quality.

In the proposed body sensor network, fraction *α* of the spectrum slot (0 ≤ *α* ≤ 1) is used for the primary transmission from PT to PR. Furthermore, this fraction *α* of the spectrum slot is divided into sub-slot *β* and sub-slot 1 − *β* in the time domain (0 ≤ *β* ≤ 1), where *α* and *β* are the parameters dynamically selected by the primary transmitter. The first sub-slot is the duration *αβ* unit time and is dedicated to the transmission of PT to all cooperative transmitters ST*_i_* in subset *S* (Figure 1a); the second sub-slot is the duration *α*(1 − *β*) unit time and in this sub-slot, all cooperative relays in subset *S* cooperatively transmit data to PR (Figure 1b). The remaining 1 − *α* of the spectrum slot is granted to the secondary transmitter to access the wireless channel and transmit data for secondary system. In this fraction of slot, *k* secondary transmitters in set *S* access the channel in Orthogonal Frequency Division Multiplexing (OFDM) mode (Figure 1c). To achieve the opportunity of accessing the spectrum, the cooperating secondary users will relay the primary user's data as return, which is called payment in this paper. It includes the bandwidth and the energy used by secondary users to transmit the primary user's data. To simplify the cooperative system model, the uniform variable *c_i_* (0 ≤ *c_i_* ≤ *c_max_*) is used to evaluate the payment of secondary user *i*, where *c_max_* represents the payment that the secondary user affords at most.

For simplicity, there are several assumptions for the mechanism as follows:

- The selected secondary users access the channel by OFDM, and hence there exists no interference among channels.
- The primary users have chosen the cognitive relays set *S* previously, including *k* pairs of secondary users.
- There is a predefined traffic requirement transmission rate *R_0_* for the primary transmission pair, and no traffic requirement is imposed for the secondary network. Each secondary link accesses the channel and transmits data as much as possible in a best-effort manner.
- There is no power control, and both primary transmitter and secondary transmitters are transmitting at a fixed power level.

All the meanings of the used variables are shown in Table 1. The different channel transmission rate in the system can be calculated according to the Shannon definition. The transmission power of the primary user is *P_p_* and secondary users' are *P_s_*. In addition, we let *h_ps,i_* denote the channels between the PT and the secondary relay ST*_i_*, *h_p_* denote the channel between the PT and PR, and *h_sp,i_* denote the channels between the ST*_i_* and the PR, respectively.

Thus, the transmission rate of each link can be calculated as follows: firstly, the situation that the primary user chooses direct transmission without cooperation is considered (*i.e.*, the traditional communication model). The transmission rate of primary user without cooperation *R*_d_ can be calculated directly based on the Shannon theorem, *i.e.*,

(1) $$R_{d}=\alpha \log_{2}\left(1+\frac{P_{P}{\left|h_{p}\right|}^{2}}{n_{0}}\right)$$

Secondly, in the case of cooperative transmission as shown in Figure 1, in the first phase *β*, the transmission rate of the primary user to the SU_i_, is given by:

(2) $$R_{ps,i}=\alpha \log_{2}\left(1+\frac{P_{P}{\left|h_{ps,i}\right|}^{2}}{n_{0}}\right)$$

In the second time phase 1 − *β*, PT and the selected ST*_i_* transmit data to the PR through the respective independent channels. At the destination, we assume the signal from the PT and the forward signal from the ST*_i_* are jointly decoded using maximum-ratio combining (MRC) [17].

MRC is a key technology of the physical layer in WLAN 802.11n, targeting on improving the signal quality of receivers. The basic principle of MRC is to receive same signals by using multiple antennas at the destination. Therefore, the signals would transmit over several channels. Since the probability of simultaneous poor quality of multi-path transmission are small, the weighted sum of signals received from all channels could be obviously improved. The resultant effective signal-to-noise ratio (SNR) at the destination is the sum of the SNR in the communication link between the PT and the PR and between the ST*_i_* and the PR. When the primary user has chosen the strategy *α* under the active cooperation-aware mechanism in this paper, the transmission rate *R_sp_* achieved by the primary user can be calculated as:

(3) $$R_{SP}=\alpha \log_{2}\left(1+\frac{P_{P}{\left|h_{p}\right|}^{2}}{n_{0}}+\underset{i\in S}{\text{∑}}\frac{P_{P}{\left|h_{ps,i}\right|}^{2}}{n_{0}}\right)$$

In this paper, the decode-and-forward (DF) type relaying is utilized. Thus, the overall transmission rate *R_p,i_* achieved by PR is equal to the minimum value of the two stages given by:

(4) $$R_{p,i}=\min\left\{R_{ps,i},(1-\beta )R_{sp,i}\right\}$$

According to the known analysis [14], the overall transmission rate *R_p,i_* could achieve the best possible optimization when the rates of two stages are equal, *i.e.*,

(5) $$\beta R_{ps,i}=(1-\beta )R_{sp,i}$$

Since the selected secondary users access the channel using OFDM, the interference can be ignored. Secondary users in subset *S* are classified into several categories according to the channel state information, including the distance to the primary receiver and the requirement of cooperative power. Accordingly, the weighting factor *ω_i_* is added to secondary users' payment *c_i_*, which increases with the channel state information and gets bigger with the decrease of the distance between the secondary transmitter and the primary receiver. Therefore, the bandwidth achieved by secondary users change with the payment *c_i_* they provide to the primary users and can be defined as follows:

(6) $$W_{S,i}=\frac{\omega_{i}c_{i}}{\underset{i}{\text{∑}}\omega_{i}c_{i}}\left(1-\alpha \right)$$

Then, the rate *R_S,i_* of secondary users SU*_i_* to transmit their own data on the achieved bandwidth *W_S,i_* is defined as follows:

(7) $$R_{S,i}=W_{S,i}\log\left(1+\frac{P_{S}{\left|h_{S,i}\right|}^{2}}{n_{0}}\right)$$

where *h_S,i_* is the channel between the ST*_i_* and SR*_i_*. The tradeoff could be immediately observed. The more *c_i_* the secondary user paid to the primary user means the more *W_S,i_* gets released to secondary users. It shows that the transmission *R_S,i_* is improved as well. Thus, secondary users have to determine how much to pay for the released spectrum.

### 2.2. Utility Function Design

#### 2.2.1. The Primary User's Utility

According to the model analyzed above, the primary utility function *U_p_*(*α,β*) is defined to be the weighted sum of the utility function of primary user's transmission rate and the revenue it collects from the secondary relays:

(8) $$U_{p}(\alpha ,\beta )=\omega_{p}C(R_{p,i}(\alpha ,\beta ))+\underset{i}{\text{∑}}\omega_{i}c_{i}$$

where *ω_P_* is the equivalent revenue per unit data rate utility that contributes to the predefined overall utility. *R_P_*(*α*,*β*) represents the maximum achievable transmission rate of the primary user. *C*(*R_P_*(*α*,*β*)), the satisfactory utility of the primary users with respect to their data rate, is defined as follows [18]:

(9) $$C(R_{p,i}(\alpha ,\beta ))=1-e^{-\alpha \lambda }$$

where *a* is the satisfaction factor (*a* >; 1), and *λ* is the ratio of the achieved rate to requirement rate, referring to the proportional fairness of resource allocation and defined as *λ* = *R_P_*(*α*,*β*)/*R_0_* . Here *R_0_* is the primary user's traffic requirement.

**Figure 2.**
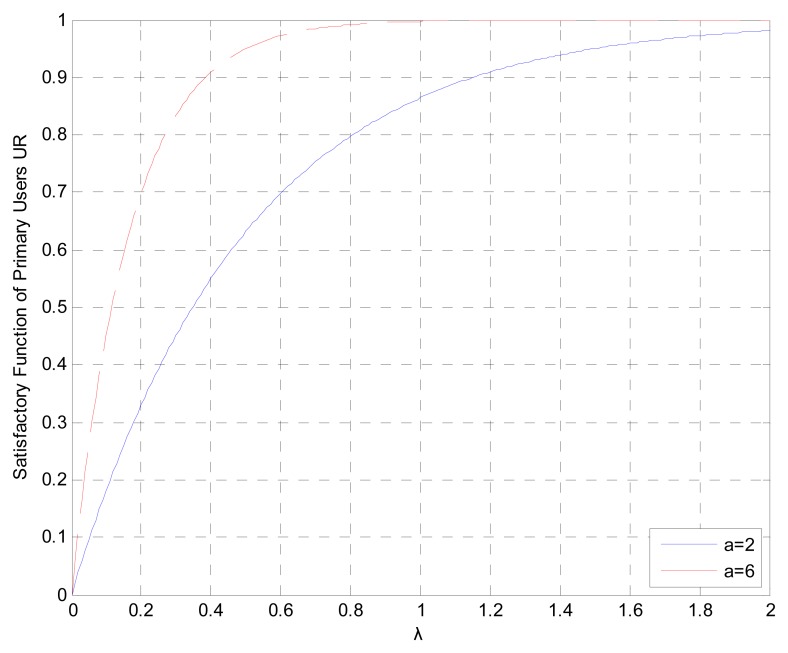
The relationship with different satisfactory factor a between the satisfaction function of the primary user and the ratio of the transmission rate to the traffic requirement λ.

The mathematical relationship between the satisfactory function of primary users and the achieved rates *versus* their requirement rates ratio, *λ*, is shown in Figure 2. The satisfaction function increases with *λ* and gets close to the transmission requirement. The increasing rate will slow down when *λ* becomes bigger. For a larger *a*, the satisfaction function of the primary user will increase faster.

#### 2.2.2. Secondary Users' Utility

The secondary users' target is to maximize the transmission rate of their own data under a reasonable payment scheme. The utility function *U_S,i_*(*c_i_*) of each secondary user is defined to be its achieved transmission rate in equivalent revenue minus the payment it makes to the primary user, *i.e.*,

(10) $$U_{S,i}(c_{i})=\omega_{s}R_{S,i}-c_{i}=\omega_{s}\frac{\omega_{i}c_{i}}{\underset{i}{\text{∑}}\omega_{i}c_{j}}(1-\alpha )\log\left(1+\frac{P_{S}{\left|h_{S,i}\right|}^{2}}{n_{0}}\right)-c_{i}$$

where *ω_s_* is the equivalent revenue per unit transmission rate contributed to the overall utility. As secondary users work in a best effort manner, and no requirement is imposed on their transmission, the utility functions are linear with the transmission rates they are able to achieve, which are proportional to the payment they are going to pay.

### 2.3. Game Problem Formulation

According to the utility function designed above, considering the different priority of primary and secondary users, the Stackelberg game theory is used to model the two optimization problems, where the time-spectrum allocation strategy is decided by the primary user and the optimal payment choice of secondary users.

Firstly, the primary user decides the time-spectrum allocation strategy by choosing the parameter *α* and *β* to improve their transmission rate with secondary users' assistance and to get extra revenue from secondary users given by:

(11) $$\left(\alpha^{*},\beta^{*})=\underset{0<\alpha <1,0<\beta <1}{\arg\max}U_{p}(\alpha ,\beta \right)$$

After the time-spectrum allocation of the primary user is decided, the selected secondary users would compete with each other to bid up the released spectrum, which intends to select an optimal payment c_i_* to maximize secondary users' transmission rate:

(12) $$c_{i}^{*}=\underset{i\in S,0\leq c_{i}\leq c_{\max}}{\arg\max}U_{S,i}(c_{i})$$

## 3. Stackelberg Game based Optimal Strategies

Taking the selfishness of both primary and secondary users into consideration, to simultaneously optimize both primary and secondary users' utilities is obviously impossible. In order to find a balanced strategy for both primary and secondary users, the above active cooperation-aware mechanism has been modeled as a typical Stackelberg two-stage single-leader-multi-follower game, where the primary user is regarded as the leader of the game and optimizes its strategy based on the knowledge of the effects of its decision on the behavior of the follower, *i.e.*, the secondary user. The balanced strategy of both primary and secondary users could be calculated by solving this game problem, and the utility of primary and secondary users could be jointly improved.

The Stackelberg leader-follower game is suited for active-aware mechanism body sensor networks using cognitive radio. For example, in the body sensor networks, the primary users, the implant nodes, can use the first stage of Stackelberg game to decide the parameter α and β, that means to decide the spectrum and time slot allocated to secondary users. Once the used spectrum and time slot for secondary users are determined, the secondary user, all kinds of wearable gauges, will use the second stage of the Stackelberg game to decide the payoff to primary users. In fact, the payoff is the application data that secondary users help the primary users to transmit. The leader-follow structure of the Stackelberg game allows the primary users to actively launch the cooperation between primary users and secondary users.

In this section, backward induction is used to deal with the optimization problem mentioned in Equations (11) and (12). It is an approach to solve the equilibrium of dynamic games with sequential actions, such as Stackelberg game where followers move after the leader to make a decision. Like the model described in Section 2, both the primary user and secondary users are rational and selfish. Firstly, assuming the primary user has determined the optimal allocation strategy, secondary users' best response function to the strategy is analyzed. After that, substituting the best response function of secondary users for the primary user's utility function, we can get the optimal primary user's time-spectrum allocation strategy.

### 3.1. Secondary Users Payment Optimal Strategy

Assuming *α*, *β* and *S* are decided by the primary user, several secondary users in the cooperative relay set *S* compete with each other for the limited 1 − *α* bandwidth to maximize its own utility by selecting its payment, forming a non-cooperative payment selection game (NPG) G = [*S*, {*C_i_*}, {*U_i_*(·)}], where *S* is the player set selected by the primary user, *C_i_* is the strategy set, and *U_i_*(·) is the utility function of user *i*. In order to solve the optimal payment strategy of secondary users, one has to prove that there exists a unique Nash equilibrium, which can be defined as follows:

**Definition 1 (Best response):**
  Player *i*'s response to be the strategy profile *s_-i_* is a mixed strategy s_i_* ∈ *S_i_* such that *U_i_*(s_i_*, *s_-i_*) ≧ *U_i_*(*s_i_*, *s_-i_*) for all strategies *s_i_* ∈ *S_i_*.
**Definition 2 (Nash equilibrium):**
  The strategy profile *s* = (*s_1_*, *s_2_*, …, *s_n_*) is a Nash equilibrium if, for all agents *i*, *s_i_* is a best response to *s_-i_* [19]. Then the existence of the optimal strategy is analyzed on the basis of convexity. In order to prove the uniqueness of the optimal strategy, it has to show that the best response function of secondary users is a standard function.

### 3.1.1. Analysis of the Existence of the NE

#### Theorem 1

*A Nash equilibrium exists in the Stackelberg game G = {S, {C_i_}, {U_S,i_(·)}} if for all i ∈ S, the following conditions (1) and (2) are satisfied:*

(1) *The game strategy set C_i_ is a nonempty, convex and compact subset of some Euclidean space*.
(2) *The game utility function U_S,i_(·) is continuous and convex in C_i_*.

#### Proof of Theorem 1

In this paper, it is evident that the strategy set *C_i_*(0 ≤ *c_i_* ≤ *c_max_*, *c_i_* ∈ *C_i_*) is a non-empty closed set and a compact convex set as well. Thus it is evident that the utility function *U_S,i_*(*c_i_*) of secondary users is continuous in the strategy set *C_i_*:

(13) $$U_{S,i}(c_{i})=\omega_{s}R_{S,i}-c_{i}=\omega_{s}\frac{\omega_{i}c_{i}}{\underset{i}{\text{∑}}\omega_{i}c_{j}}(1-\alpha )\log\left(1+\frac{P_{S}{\left|h_{S,i}\right|}^{2}}{n_{0}}\right)-c_{i}$$

As for the quasi-concavity *U_S,i_*(*c_i_*), by differentiating *U_S,i_* with respect to *c_i_*, we have:

(14) $$\frac{\partial U_{S,i}}{\partial c_{i}}=\omega_{s}\frac{(1-\alpha )\log\left(1+\frac{P_{S}{\left|h_{S,i}\right|}^{2}}{n_{0}}\right)\omega_{i}\left(\underset{j\neq i}{\text{∑}}\omega_{j}c_{j}\right)}{{\left(\underset{j}{\text{∑}}\omega_{j}c_{j}\right)}^{2}}-1$$

Furthermore, the second order derivative of *U_S,i_* can be calculated as follows:

(15) $$\frac{\partial U_{S,i}}{\partial c_{i}}=\frac{-2\omega_{s}(1-\alpha )\log\left(1+\frac{P_{S}{\left|h_{S,i}\right|}^{2}}{n_{0}}\right)\omega_{i}\underset{j\neq i}{\text{∑}}\omega_{j}c_{j}}{{\left(\underset{j}{\text{∑}}\omega_{j}c_{j}\right)}^{2}}<0$$

As previously shown above, the second order derivative of *U_S,i_* with respect to *c_i_* is less than 0, which means that the utility function of secondary users in the proposed non-cooperative game is quasi-concave in *c_i_*. Therefore, there exists the Nash equilibrium in the game. This concludes the proof.

### 3.1.2. Analysis of the Uniqueness of the NE

#### Theorem 2

The Nash equilibrium of the non-cooperative game G = {S, {C_i_}, {U_S,i_(·)}} is unique.

#### Proof of Theorem

According to Theorem 1, the existence of the Nash equilibrium in the game has been proved. Thus the key issue is to prove that the best response c_i_* is a standard function [20]. By taking the derivative of the U_S,i_ to c_i_ ,and equating it to zero, we have:

(16) $$\frac{\partial U_{S,i}}{\partial c_{i}}=\omega_{s}\frac{(1-\alpha )\log\left(1+\frac{P_{S}{\left|h_{S,i}\right|}^{2}}{n_{0}}\right)\omega_{i}\left(\underset{j\neq i}{\text{∑}}\omega_{j}c_{j}\right)}{{\left(\underset{j}{\text{∑}}\omega_{j}c_{j}\right)}^{2}}-1=0$$

Solving the above-mentioned equation for *c_i_*, the optimal payment c* could be denoted as:

(17) $$c_{i}^{*}=\sqrt{\omega_{s}\omega_{i}^{-1}(1-\alpha )\log\left(1+\frac{P_{S}{\left|h_{S,i}\right|}^{2}}{n_{0}}\right)\underset{j\neq i}{\text{∑}}c_{j}}-\underset{j\neq i}{\text{∑}}c_{j}$$

The best response function c_i_* is a positive, monotonic and extensible standard function. Therefore the Nash equilibrium in the game G = {*S*, {*C_i_*}, {*U_S,i_*(·)}} is unique. This concludes the proof.

Taking for *i* = 1, 2, …, *k*, to solve the equations with *k* unknowns, it results in the unique Nash equilibrium, *i.e.*, the best response function of secondary users' payment given by:

(18) $$c_{i}^{*}=\omega_{s}\omega_{i}^{-1}(1-\alpha )(k-1)\left[\underset{j\in S}{\text{∑}}\frac{1}{\log\left(1+\frac{P_{S}{\left|h_{S,j}\right|}^{2}}{n_{0}}\right)}-\frac{k-1}{\log\left(1+\frac{P_{S}{\left|h_{S,j}\right|}^{2}}{n_{0}}\right)}\right]{\left(\underset{j\in S}{\text{∑}}\frac{1}{\log\left(1+\frac{P_{S}{\left|h_{S,j}\right|}^{2}}{n_{0}}\right)}\right)}^{-2}$$

Therefore, the optimal secondary payment strategy can be achieved.

## 3.2. Primary User Allocation Strategy

As shown in the previous section, the primary user may choose the arbitrary parameters *α* and *β* to determine the time-spectrum allocation in an active cooperation. Based on the analytical result of secondary users' payment selection game, the primary user (the leader of the game) can optimize its strategy (*α*, *β*, *S*) in order to maximize its revenue, being aware that its decision will affect the strategy selected by the followers (secondary users).

### 3.2.1. The Optimal Sub-Time-Slot Strategy

Firstly, substituting Equation (19) for Equation (11), the utility *U_p_*(*α*,*β*) of the primary user is derived as:

(19) $$U_{p}(\alpha ,\beta )=\omega_{p}C(R_{p}(\alpha ,\beta ))+\frac{\omega_{s}(1-\alpha )(k-1)}{\underset{j}{\text{∑}}\left(1/\log\left(1+\frac{P_{S}{\left|h_{S,j}\right|}^{2}}{n_{0}}\right)\right)}$$

The overall transmission rate could achieve optimization when the rates of two stages are the same. However, in the primary broadcast data to the selected secondary relay in phase *β* over multi-relay cooperative communication systems, to make the ST*_i_* decode successfully, the transmission rate *R_PS_*(*S*) depends greatly on the worst channel rate given by:

(20) $$R_{PS}=\min_{i\in S}\left\{R_{ps,i}\right\}=\min_{i\in S}\left\{\alpha \log_{2}\left(1+\frac{P_{S}{\left|h_{SP,i}\right|}^{2}}{n_{0}}\right)\right\}$$

In the second phase 1 − *β*, since both the primary user and the ST*_i_* transmit the data to the PR cooperatively, the transmission rate can be calculated by Equation (7). Let β* denote the optimal sub-time-slot strategy selected by the primary user. According to Equation (8), when there is a constraint:

(21) $$\beta R_{PS}=(1-\beta )R_{SP}(S)$$

the optimal sub-time-slot strategy β* can be calculated as:

(22) $$\beta^{*}=R_{SP}(S)/\left(R_{SP}(S)+R_{PS}(S)\right)$$

Therefore, the maximum overall cooperation transmission rate *R_p_*(*α*,*β*) of the primary user could be calculated as:

(23) $$R_{p}(\alpha ,\beta )=\beta^{*}R_{PS}(S)$$

### 3.2.2. The optimal sub-spectrum-slot strategy

Let α* denote the optimal sub-spectrum-slot strategy selected by the primary user. Taking Equation (8) for the utility function of the primary user *U_P_*, and calculating the first order derivate of *U_P_* to *α*, we obtain:

(24) $$\frac{\partial U_{p}}{\partial \alpha }=\omega_{p}Ae^{-\alpha A}-B$$

where:

(25) $$A=\alpha \beta^{*}\left(\min_{i\in S}\left\{\log_{2}\left(1+\frac{P_{S}{\left|h_{ps,i}\right|}^{2}}{n_{0}}\right)\right\}\right)/R_{0}$$

and:

(26) $$B=\omega_{s}(k-1)/\underset{j}{\text{∑}}\left(1/\log\left(1+\frac{P_{S}{\left|h_{S,j}\right|}^{2}}{n_{0}}\right)\right)$$

The optimal spectrum strategy α* is thus achieved by assigning the first order derivative to be 0, *i.e.*,

(27) $$\alpha^{*}=-\ln\left[B/(\omega_{s}A)\right]/A$$

Therefore, the optimal strategy of both primary and secondary users is derived. Under the above-mentioned analyses, we propose an algorithm for the primary user and secondary users to achieve their optimal strategies, as shown in Algorithm 1.


**Algorithm 1** Stackelberg Game based Optimal Strategies
1. All secondary users inform the primary user to join an active cooperation. The set is *S*.2. The primary user decide the initial strategies *α*^1^ and *β*^1^ according to its data length, and then inform all secondary users in *S* of the initial strategies.3. All secondary users in *S* calculate the initial optimal payment strategies *c_i_*^1^ in Equation (18) according to the primary user's initial strategies, and feedback these to the primary user.4. According to secondary users' strategies, the primary user calculates the optimal payment strategies *α^n^* and *β^n^* with Equations (22) and (27).5. The primary user compares the current strategies *α^n^* and *β^n^* with the last strategies *α^n^*^−1^ and *β^n^*^−1^. If *α^n^*–*α^n^*^−1^= *ε* and *β^n^*–*β^n^*^−1^= *ε* (*ε* is a small number, for example: 0.001), the primary user will no longer update the strategies which means it will use *α^n^*^−1^ and *β^n^*^−1^ as the fixed strategies. Otherwise it has to change strategies to *α^n^* and *β^n^*. Then the primary user sends the strategies to *S*.6. All secondary users in *S* calculate their optimal payment strategies *c_i_^n^* in Equation (18) according to the primary user's strategies.7. All secondary users in *S* compare their current strategies *c_i_^n^* with the last strategies *c_i_^n^*^−1^. If *c_i_^n^* –*c_i_^n^*^−1^= *ε*, SU will no longer update strategies which means they use *c_i_^n^*^−1^ as the fixed strategies. Otherwise they change strategies to *c_i_^n^*.8. If the primary user and all secondary users in *S* do not change their strategies in the last round, the game reaches the Nash equilibrium and then end. Otherwise all secondary users in *S* send the strategies to the primary user and go to step 4.


## 4. Simulation Results and Analysis

In this section, a real body sensor network is taken as a simulation scene to validate the performance of the proposed active cooperation-aware mechanism. The body sensor networks consists a pair of primary users and *K* pairs of secondary users (*K* = 6). The primary user is an implant node used to measure blood glucose levels, which uses the licensed spectrum for the medical implant communications service (MICS) 402–405 MHz band. Six secondary users are wearable gauges to measure the blood pressure, the breath rate, pulse rate, blood oxygen, body posture and electrocardiography. These wearable gauges use the ISM spectrum to transmit the sensed data.

To reflect the distance affection to cooperation of primary user and the secondary users, the locations of all secondary user are approximately the same normalized distance *d*(0 <; *d* <; 1) from the PT, 1-*d* from the PR. The PT then chooses *k* secondary users as cooperation relays (2 ≤ *k* ≤ *K*). Assuming that channel gain is *h_ij_* = *K*(*d_0_*/*d_ij_*)*η*, where *K* = *d_0_* is the normalization constant and *η* represents the SNR in the channel. We take for *K* = *d_0_* = 1 and *η* = 3 for simplicity. Thus the average channel gain between the primary user and secondary users is *E|h_PS,i_|^2^* = 1/*d^η^* and *E|h_SP,i_|^2^* = 1/(1 − *d*)*^η^*. For the cognitive network, the channel gain at the transmitter and the receivers of secondary users are *E|h_S,i_|^2^* = 0.8. In addition, we take for *ω_P_* = 0.3, *ω_s_* = 0.15, and *ω_i_* = 1/(1 − *d*)*^η^*.

### 4.1. Secondary User Relay Power Simulation

**Figure 3.**
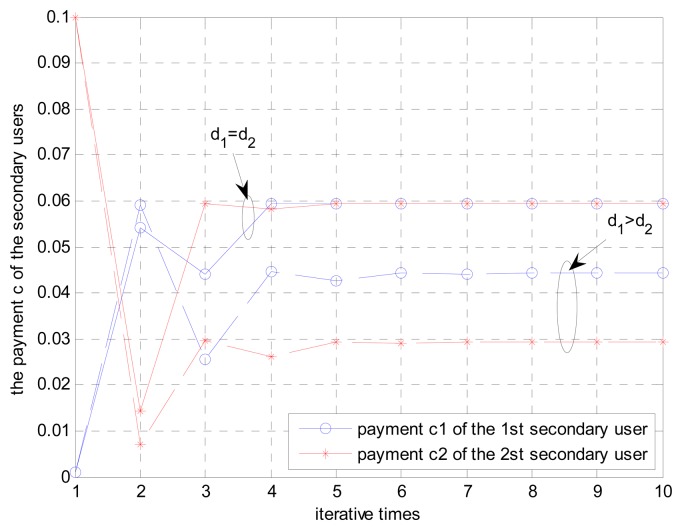
Observation of convergence of relay power c of secondary users for d_1_ = d_2_ and d_1_ >; d_2_, where d_1_ is the distance between the first secondary user and primary transmitter and d_2_ is the distance between the secondary user and primary transmitter.

Firstly, the relay power of secondary users is verified. As shown in Figure 3, different channel state information of secondary users leads to different convergences where secondary users take for k = 2. In Figure 3, the selected two symmetrical secondary users have the same distance *d* from the primary user but different distances from the primary user, respectively. It shows that the convergence to the NE occurs after five iterations of both conditions, although the channel state information and payment weighing factors are different. Thus the convergences of two secondary users are different with five iterations.

### 4.2. Primary User Allocation Simulation

**Figure 4.**
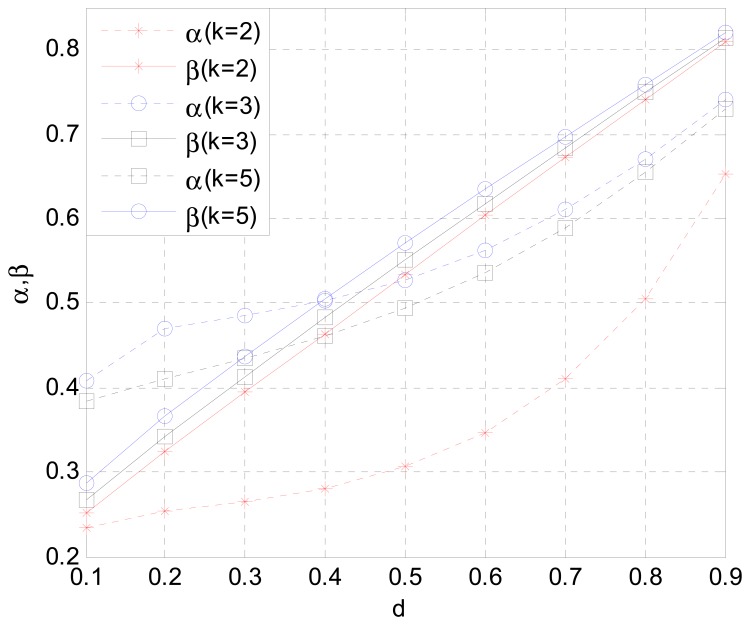
Optimal time allocation strategy α* and optimal bandwidth strategy β* versus distance *d* between secondary user and primary transmitter with different numbers of relays.

As shown in Figure 4, the optimal parameters α* and β* are changed with the normalized distance of all secondary users' locations to PT *d* under various numbers of secondary relays. With the increased distance *d*, the broadcast transmission rate from PT to ST_i_ decreases. However, the cooperative transmission rate from ST_i_ to PR is increased. To receive a certain amount of data and forward the same amount, more time is needed for the first broadcast stage and less is needed for the second cooperation stage. Therefore, *β* increases when the normalized distance *d* becomes larger, which agrees with the analysis result in Equation (23). In addition, *α* is also increased when the unified distance becomes larger, but with a smaller increasing rate, which complies with the analysis result of the balance of interests to the Stackelberg game.

**Figure 5.**
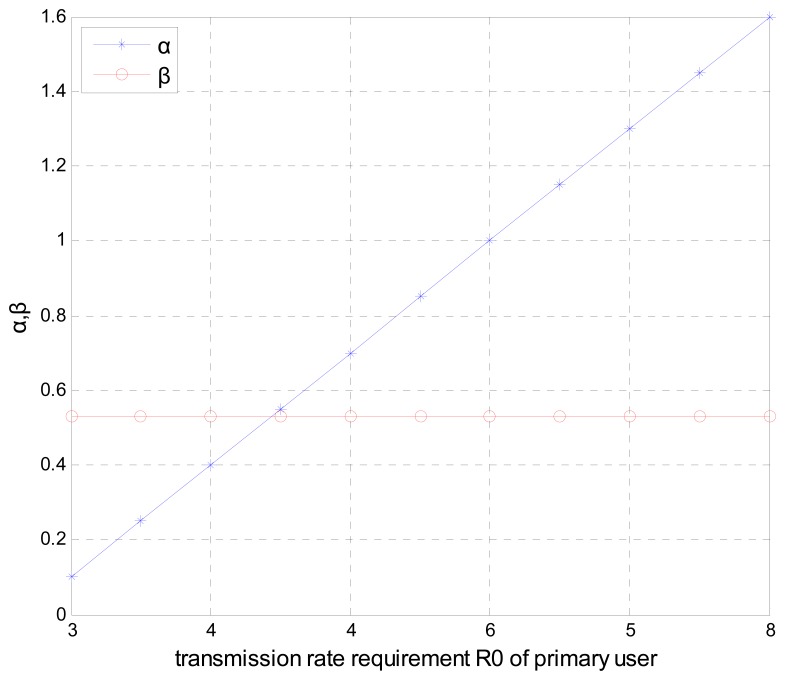
Optimal time allocation strategy α* and optimal bandwidth strategy β* versus the transmission rate R_0._

Figure 5 shows the primary user"s optimal strategy α* and β* with user's transmission rate (the normalized distance *d* = 0.3, and the number of the secondary relays *k* = 5). When the primary user's transmission rate *R_0_* is increased, the bandwidth strategy parameter α* goes up linearly, since the larger *R_0_* requires more time spent on transmission of the primary user's data. While the optimal bandwidth strategy β* remains independent of the transmission rate requirement *R_0_* and stays constant, this simulation result also complies with the analyzed result given in Section 3.

### 4.3. User's Utility Analysis

**Figure 6.**
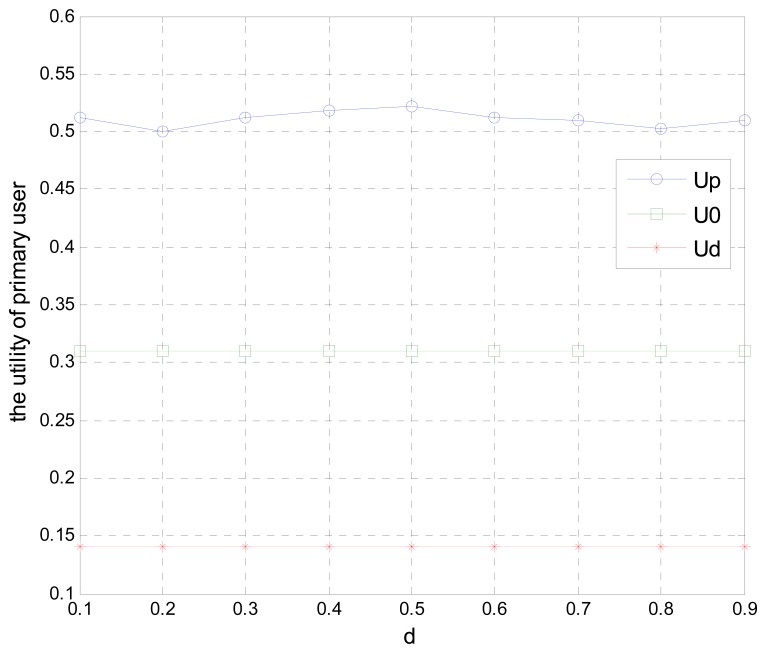
The primary user's utility function of different schemes, where *U_P_* denotes the utility function of the proposed active-cooperation scheme, *U_0_* denotes the primary user's utility function where all the bandwidth is given to secondary users, and *U_d_* denotes the utility function all the bandwidth is only used to transmit the primary user' own data without cooperating with secondary users.

Figure 6 shows the relationship between the primary user's utility and the normalized distance *d* of three different schemes, where *U_P_* denotes the utility function of the optimal scheme, in which the primary user cooperates with secondary users under the active cooperative scheme, *U_0_* denotes the primary user's utility function when *α* = 0, which implies that all of the primary user's bandwidth is given to secondary users to receive payment without sending any their own data and achieves no transmission rate, and *U_d_* denotes the utility function of the primary user when no cooperation exists and all the bandwidth is only used to transmit their own data without optimizing the communication properties by leasing spectrum to secondary users. As shown in Figure 6, the utility function *U_P_* under the active cooperative scheme is superior to the utility functions *U_0_* and *U_d_*.

**Figure 7.**
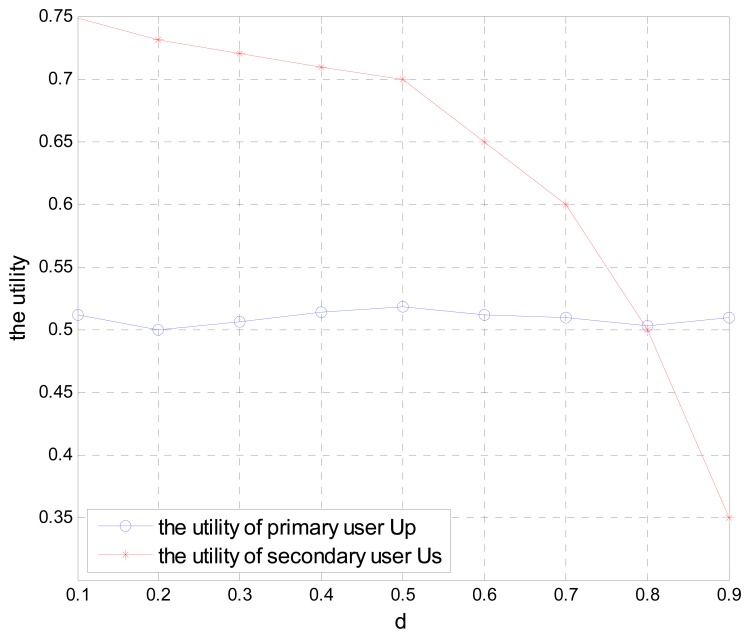
Utility of the primary user and secondary users with different distance.

As shown in Figure 7, when the cooperative scheme is used, the utility function of secondary user decreases as the normalized distance of all secondary users' locations to PT *d* increases. In this case, the leased spectrum and the access opportunities of secondary users are both increased. Since the utility of the primary user consists of two parts, *i.e.*, the achieved transmission rate and the payment from secondary users, as shown in Equation (11), the cooperative users adaptively adjust their own payment in the Stackelberg game and cause the utility function of the primary user to have tiny fluctuations. Thus in the Stackelberg game, the primary user's long distance communication is guaranteed by activating the secondary users' cooperation.

**Figure 8.**
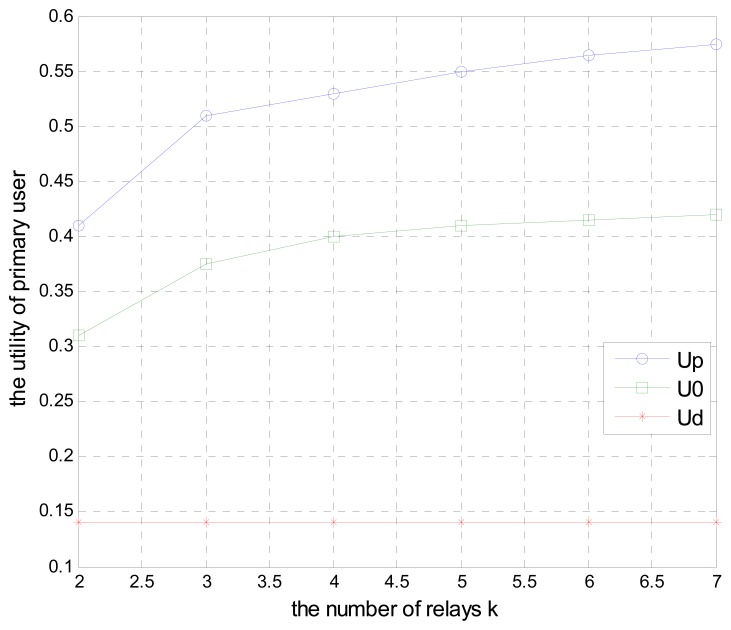
The primary user's utility function of different schemes with different number of relays.

Figure 8 reflects the relationship between the utility function and the number of secondary relays under three different schemes as analyzed above. When the distance between the primary user and secondary users is *d* = 0.3, as shown in the figure, *U_d_* remains constant when the primary user has not cooperated with secondary users by leasing spectrum and transmits their data directly to the primary receiver. When all of the primary user's bandwidth is given to secondary users, the utility *U_0_* goes up as the number of the secondary relays increases. Similarly, the utility *U_P_* under the cooperative scheme also increases when the number of secondary users increases, and is better than other two utilities. In addition, *U_0_* and *U_P_* remain stable when the number of secondary relays reaches to a certain threshold due to the limited resources become saturation.

## 5. Conclusions

In this paper, we investigate the cognitive radio technology in body sensor networks, which consists of body-implanted sensor nodes and wearable sensor nodes to monitor human body signals. The body sensor network requires high real-time performance and it is sensitive to energy consumption, especially for body-implanted sensor nodes. To improve the spectrum utilization, the cognitive radio method is introduced to enhance the real-time transmission. To reduce the energy consumption, a cooperative incentive mechanism is proposed for the cognitive radio scenario. This mechanism is based on the Stackelberg game theory, using the cooperative communication technology to enhance the spectrum efficiency, network throughput and reliable communication performance. The primary user incentivizes secondary users to cooperative with it by releasing a portion of its spectrum to the selected secondary users to insure the communication continuity, and secondary users help the primary user to transmit its data as return. By formulating this mechanism as a Stackelberg game, and proving the existence and the uniqueness of the Nash Equilibrium of game, the utility function of both the primary user and secondary users are analyzed. The result of both the simulation and the analysis show that the active cooperative-aware mechanism in this paper could converge to the unique NE, and both the primary user and secondary users in body sensor networks can achieve better performance and utilities. The proposed scheme can be extended to more complex scenarios in which multiple primary users may transmit concurrently, and the interference among primary users in the body sensor networks can be considered as one of the power adjusting parameters in future research.

## Figures and Tables

**Table 1.** The meanings of the variables.

**Variables**	**Meaning**
*P_p_*	transmission power of the primary user
*P_s_*.	transmission power of secondary users
*h_ps,i_*	channels between the PT and the secondary relay ST*_i_*
*h_p_*	the channel between the PT and PR
*h_sp,i_*	the channels between the ST*_i_* and the PR
*R*_d_	the transmission rate of primary user without cooperation
*R_ps.i_*	the transmission rate of the primary user to the SU_i_
*R_sp_*	the transmission rate of all SUs to PR
*R_p,i_*	the overall transmission rate achieved by PR
*c_i_*	the payment of SU*_i_*
*W_S,i_*	the bandwidth achieved by secondary users change with the payment *c_i_*
*R_S,i_*	the rate of secondary users SU*_i_* to transmit their own data on the achieved bandwidth *W_S,i_*
*U_p_*(*α*,*β*)	the primary utility function
*C*(*R_p,i_*(*α*,*β*))	the satisfactory utility of the primary users
*λ*	the ratio of the achieved rate to requirement rate, referring to as the proportional fairness of resource allocation and defined as *λ* = *R_P_*(*α*,*β*)/*R_0_*
*U_S,i_*(*c_i_*)	the utility functionof each secondary user
*d*	the normalized distance of all secondary users' locations to PT
